# Atmospheric emission assessment for LNG carriers: a state-of-the-art semi-empirical methodology

**DOI:** 10.1007/s11356-025-36037-8

**Published:** 2025-02-10

**Authors:** Carlos Gonzalez Gutiérrez, Álvaro Herrero Martínez, Emma Diaz Ruiz de Navamuel, Andrés Ortega Piris, Beatriz Blanco Rojo

**Affiliations:** Coastal and Ocean Planning and Management R+D Group, University of Cantabria, Germán Gamazo nº 1, 39004 Santander, Cantabria Spain

**Keywords:** LNG, Atmospheric emissions, Fuel consumption

## Abstract

This research establishes an extensive set of algorithms for the precise evaluation of fuel consumption across a range of engine types and systems onboard LNG carriers (LNGCs). The principal objective of this research is the development of a modern, universally applicable, and refined methodology tailored to LNGCs. This methodology encompasses the comprehensive calculation of both fuel consumption and atmospheric emissions, accommodating the variability in engine configurations. Furthermore, the integration of empirical data derived from 73 operational LNGCs yields a meticulously crafted operational profile. This consequential outcome of our study carries significant implications for addressing forthcoming modeling complexities specific to LNGCs.

## Introduction

In 2021, global liquefied natural gas (LNG) exports surged by 21 Mtons, continuing the upward trajectory observed in recent years, surpassing the projected LNG demand for the same year (Shell 2022). This growth was further underpinned by the expansion of both regasification and liquefaction facilities in 2021 (International Gas Union 2022). The pivotal role of LNG shipping in meeting the world’s energy needs cannot be overstated.

As of July 2022, the order book boasted 229 LNG carriers (LNGCs) scheduled for construction in the months and years ahead (TradeWinds 2022). This fleet expansion, combined with the ever-increasing global energy demands, underscores the critical role played by LNG carriers in the energy trade. Furthermore, the utilization of LNG as a marine fuel has witnessed a remarkable 30% increase, emerging as an alternative for non-LNGC vessels (Shell 2022).

**Fig. 1:**
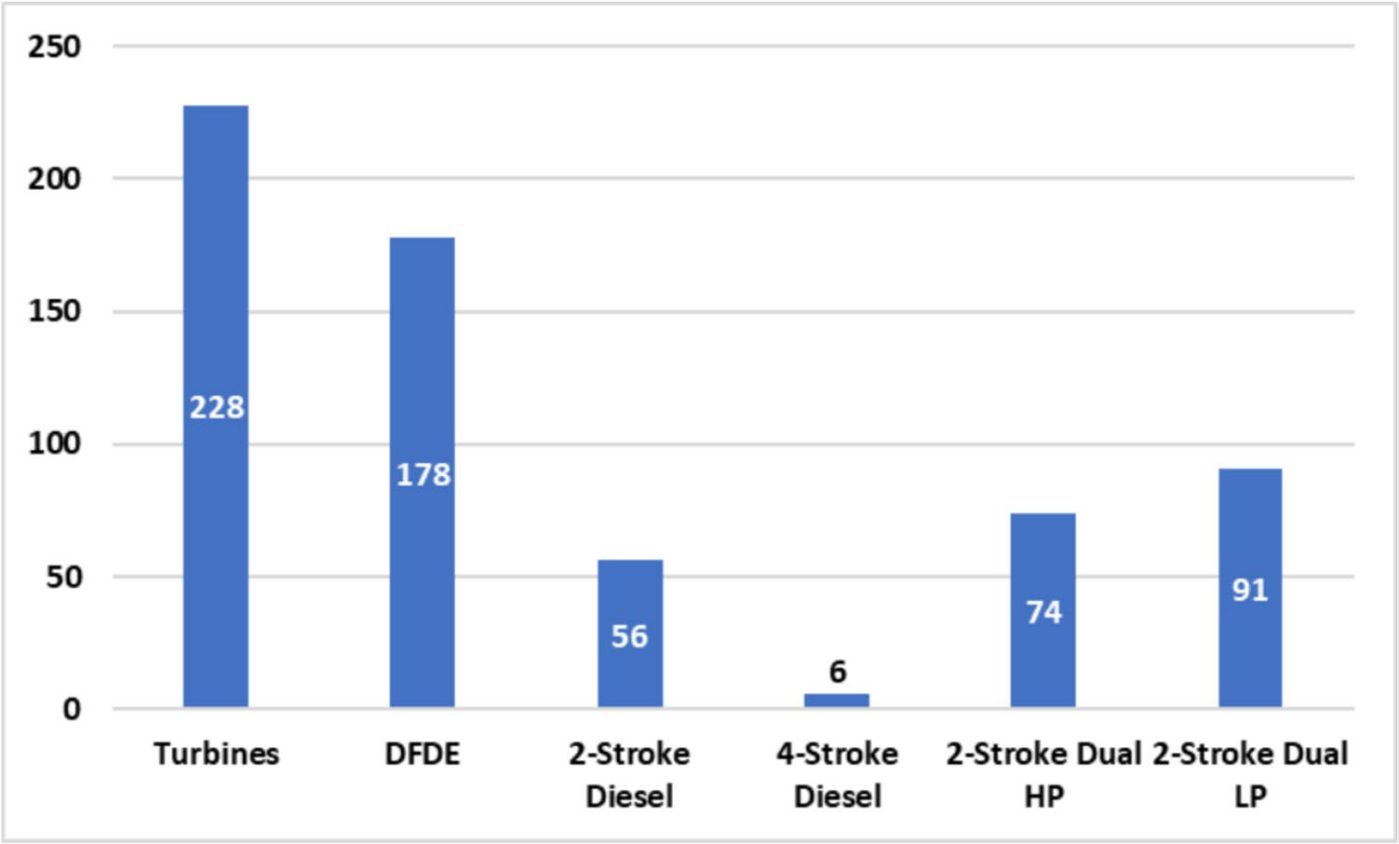
LNGC fleet in service by propulsion systems. Information Handling Services Markit 2022. *HP: gas injection at high pressure, LP: gas injection at high pressure

By August 2022, the IHS Fairplay database recorded 633 LNGCs in active service, featuring diverse propulsion systems, as illustrated in Fig. 1 (Information Handling Services Markit 2022).

Because of the expanding fleet, the total atmospheric emissions from this sector are projected to increase. The development of comprehensive ship exhaust emission inventories serves as a vital scientific foundation for the establishment of regulations aimed at curtailing ship emissions (Peng et al. 2020). It is, therefore, imperative to possess accurate methods for calculating ship emissions.

Presently, two prevailing methodologies are widely employed for assessing ship emissions: the top-down approach, which yields aggregated results with relatively low resolution but lacks specificity regarding individual shipping activities, and the bottom-up or activity-based approach, which offers high-resolution data but demands significant time and data resources (Topic et al. 2021).

One of the seminal reports in the maritime industry, the 3rd GHG Report by the International Maritime Organization (IMO) (Smith et al. 2015), delved into the bottom-up approach, which, although valuable, is subject to certain assumptions and uncertainties regarding fuel types (Ančić and Šestan 2015). Despite its contributions, this method encounters significant challenges when analyzing the “Gas Carriers” category, which aggregates both LNG and LPG carriers. These vessel types exhibit marked differences, particularly in commercial operations and propulsion systems, leading to potential inaccuracies in emission estimation (Aldous et al. 2015; Wang et al. 2015; López-Aparicio et al. 2017; Sheng et al. 2018; Zhang et al. 2018). Moreover, previous studies have often emphasized aggregated results without accounting for the operational nuances of LNGCs, thereby limiting the specificity of emission estimates. This research addresses these gaps by developing a tailored methodology for LNGCs, validated using operational data from active vessels, to achieve more accurate and precise emission modeling.

In 2020, the IMO released the 4th GHG Report (IMO 2020), introducing enhancements to the bottom-up methodology and incorporating updates in operational profiles and emission factors.

In alignment with the efforts of IMO, various stakeholders within the maritime industry are translating their ambitions for shipping decarbonization into practical guidelines. Initiatives such as the Poseidon Principles and the Sea Cargo Charter, among others, have been established to create a common, global baseline for quantitatively assessing whether portfolios align with adopted climate goals.

The primary objective of this paper is to develop a straightforward, universal, and up-to-date methodology for calculating the fuel consumption of LNGCs, considering the various engine types. This methodology will facilitate the creation of emission inventories and enable comprehensive analysis.

The model presented in this paper builds upon the activity-based approach utilized by the IMO as its starting point. It is then enriched with operational data collected from 73 LNGCs currently in service, each equipped with diverse propulsion systems. This approach allows for a more detailed and granular analysis of the various propulsion systems utilized on LNGCs, as well as an exploration of different operational profiles and fuel mixtures used. Furthermore, the model incorporates technical performance data from the propulsion systems installed on LNGCs, which is sourced from leading providers in the industry.

The subsequent sections will cover our methodology and data sources, model validation, future work, and conclusions.

## Methodology and data sources

### General overview

**Fig. 2:**
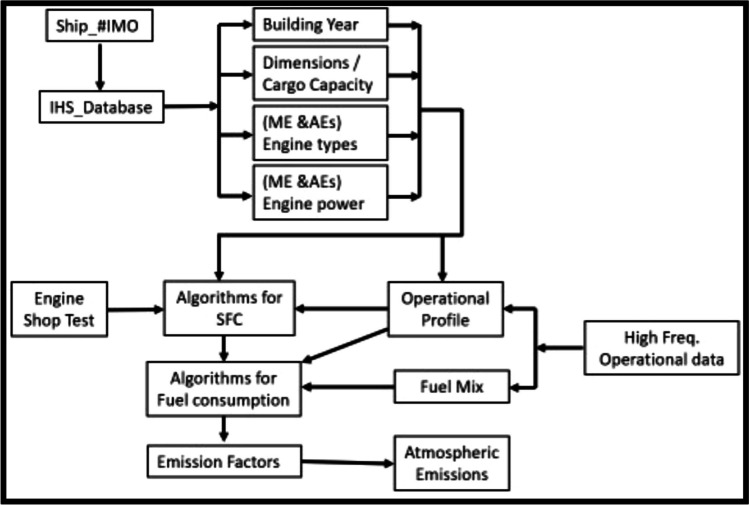
Flowchart for the proposed methodology

The proposed methodology is illustrated in Fig. 2. For LNGC with conventional 2-stroke or 4-stroke diesel engines (around 10% of the fleet), the method described in the 3rd and 4th IMO GHG Study is used (Smith et al. 2015; IMO 2020). However, for the following engine types, this work brings a novel method:

- Two-stroke dual engines with gas injection at high pressure (2MEDHP)
- Two-stroke dual engines with gas injection at low pressure (2MEDLP)
- Four-stroke dual engines with gas injection
- Steam plants

The activity-based methodology is enriched with high-frequency data obtained from a comprehensive dataset of 73 LNGCs in service, spanning the entire year 2019. This dataset is used to develop an updated operational profile tailored to each engine type. Furthermore, to ensure the development of a comprehensive method, algorithms are defined to calculate specific fuel consumption (SFC) for each engine type. These algorithms are derived from the technical performance data obtained through official shop tests for a range of main engines, auxiliary engines, and steam plants.

The subsequent subsections in this paper detail the following key elements: data sources, fuel and atmospheric emission algorithms for various fuel consumers, the operational profiles, and the emission factors used.

### Data sources

Operational profiles have been meticulously updated by utilizing the high-frequency operational data collected from 73 LNG carriers (LNGCs) in service throughout the year 2019. A comprehensive explanation of the method and the parameters employed for data processing can be found in González Gutiérrez et al.’s (2020) study.

To determine the fuel consumption of various engine types, we rely on engine shop test reports specific to each relevant engine model. In the case of steam plants, we draw upon performance sheets for the primary boilers and employ energy balances for the steam plants.

### Fuel consumption for main engine

The main engine’s fuel consumption depends on the engine power, engine load (MCR), and year of build. The MCR is affected directly by the ship’s speed. Other factors such as the ship’s load condition and weather conditions also influence the engine MCR (Jalkanen et al. 2012; Smith et al. 2015).

On LNGC, the main engines can be steam plants, diesel engines, or dual fuel engines. The latter two types may be found onboard as auxiliary engines too. Diesel engines operate using a single type of liquid fuel at a time, either RO or DO. In contrast, dual fuel engines can operate in either diesel mode or gas mode.

When operating in gas mode, dual-fuel engines require a small amount of diesel oil, referred to as “pilot fuel,” to initiate and sustain the combustion process. The quantity of pilot fuel required depends on the engine load. This consumption is typically expressed as the specific pilot fuel consumption (SPFC), which represents the amount of pilot fuel consumed per unit of engine power output during dual-fuel operation. SPFC is measured in grams per kilowatt-hour (g/kWh).

1 $${\text{FC}}_{\text{ME},i}=\sum_{f=1}^{F}\frac{({\text{SFC}}_{m,f,i}+{\text{SPFC}}_{m,f,i})\bullet {P}_{f}\bullet E{L}_{f}\bullet {h}_{f}}{{10}^{6}}$$

where ${\text{FC}}_{\text{ME},i}$ is the annual fuel consumption in tons, $i$ is the fuel type, *f* are the voyage phases, *F* is the total number of phases, $\text{SFC}$ is the SFC in g/kWh, $\text{SPFC}$ is the specific pilot fuel consumption (SPFC) in g/kWh, *m* is the engine type, *P* is the engine power for each phase as kW, $\text{EL}$ is the MCR as a decimal, and $h$ is the number of hours in operation.

The SPFC for 2-stroke and 4-stroke conventional diesel engines is zero.

For LNGC with DFDE where various 4-stroke dual engines are installed on board, the fuel consumption is calculated as shown in Eq. 2.

2 $${\text{FC}}_{\text{DDG},i}=\sum_{f=1}^{F}\frac{({\text{SFC}}_{\text{DDG},f,i}+{\text{SPFC}}_{\text{DDG},f,i})\bullet {P}_{f}\bullet {\text{EL}}_{f}\bullet \frac{{N}_{o,f}}{{N}_{a}}{\bullet h}_{f}}{{10}^{6}}$$

where ${\text{FC}}_{\text{DDG},i}$ is the annual fuel consumption for the 4-stroke dual engines in tons, $i$ is the fuel type, $f$ are the voyage phases, and *F* is the total number of phases. The terms SFC, SPFC, *P*, $\text{EL}$, and *h* retain the same definitions as introduced after Eq. 1. Additionally,* t* is the fuel type, $m$ is the engine type, ${N}_{o}$ are the number of engines in operation (Table 1), and ${N}_{a}$ is the number of engines installed on board.

**Table 1:** Number of auxiliary engines in service and load for each voyage phase. Author’s data

Voyage phase	Turbine		DFDE*		2-stroke Diesel		2-Stroke Dual	
	#AE	MCR (%)	#AE	MCR (%)	#AE	MCR (%)	#AE	MCR (%)
Normal navigation	1.30	41.4	3.53	70.7	2.76	42.1	2.17	51.9
Slow steaming	1.40	33.5	2.39	60.0	2.73	42.3	2.21	52.0
Average navigation	1.36	35.7	2.86	64.6	2.73	42.2	2.19	52.0
Maneuvring	1.67	29.1	1.50	40.4	2.86	42.1	2.45	46.4
At anchorage	1.82	29.4	1.71	23.5	2.89	44.4	2.61	46.6
At berth	1.76	35.5	1.22	28.4	2.38	34.3	2.66	47.9

^*^On ships with DFDE propulsion, dual four-stroke engines have connected alternators that produce electrical power. This electrical energy is used to supply the energy required for propulsion, and for auxiliary energy, for this reason, the MCR is higher in maneuvring, at anchorage, and at port than the other propulsion systems. However, it was observed that in LNG ships with this propulsion system, if we observe the MCR of the propulsion system in isolation (power delivered to the propeller), the MCR values were 80.5% for “Normal Navigation,” 41.4% for “Slow Steaming,” 10.2% for “Maneuvring”, 3.2% for “Anchored,” and 1.2% for “Port”

The fuel consumption of any engine depends on then the operational profile and also engine’s design parameters which influence the SFC (Winnes and Fridell 2010; Anderson et al. 2015; Jalkanen et al. 2016; Jafarzadeh and Schjølberg 2018).

The following subsections explain the developed methods to calculate the SFC for each engine and fuel type.

#### SFC for two-stroke and four-stroke diesel engines

Conventional diesel engines acting as propulsion engines are classified as slow-speed diesel (SSD), medium-speed diesel (MSD), and high-speed diesel (HSD).

The fuel type is also an important factor regarding fuel efficiency within combustion processes (Winnes and Fridell 2009). In the internal combustion process, it is estimated that the SFC is 10 g/kWh lower when the fuel in use is distillate oil (DO) instead of residual oil (RO) (European Environment Agency 2013). The base SFC of each of these engine types is shown in Table 2.

**Table 2:** SFC base for conventional diesel engines. (Adapted from Smith et al. 2015)

Year	SSD		MSD		HSD	
	RO	DO	RO	DO	RO	DO
In or before 1984	205	195	215	205	225	215
Between 1984 and 2000	185	175	195	185	205	195
In or after 2001	175	165	185	175	195	185

Units: g/kWh

The general approach assumes that the correlation between SFC and engine load follows a quadratic relationship, as demonstrated by Jalkanen et al. (2012) and Smith et al. (2015). Jalkanen et al. (2012) show that SFC for medium and large engines follows a parabolic curve, with a minimum occurring at an engine load of approximately 70–80%, which is considered the optimal operating range for fuel consumption and engine performance. This minimum is due to the balance between the engine’s efficiency at lower loads (where fuel consumption is higher) and at higher loads (where mechanical and thermal limits cause diminishing returns in efficiency). In our model, a quadratic function is used to represent this non-linear behavior, as per Eq. 3.

3 $${\text{SFC}}_{\text{CE}}={\text{SFC}}_{\text{base},m}\bullet \left(0.455\bullet {\text{EL}}_{f}^{2}-0.71\bullet {\text{EL}}_{f}+1.28\right)$$

where ${\text{SFC}}_{\text{CE}}$ is the SFC of conventional diesel engines and ${\text{SFC}}_{\text{base}}$ is the SFC base from Table 2 both in g/kWh, $m$ is the engine type, $\text{EL}$ is the MCR as a decimal, and $f$ is each voyage phase. This engine type uses only liquid fuels.

#### SFC for two-stroke dual engines with gas injection at high pressure

This engine type operates both in gas and diesel modes, and the way of calculating the SFC and SGC is shown in Eqs. 4 and 5 respectively.

4 $${\text{SFC}}_{{\text{ME}}_{\text{HP},i}}=\frac{\left(-28.79\bullet {\text{EL}}_{f}^{3} + 110.94\bullet {\text{EL}}_{f}^{2} - 110.29\bullet {\text{EL }}_{f}+ 196.12\right)\bullet 42.7}{{\text{LHV}}_{\text{actual},i}}$$

5 $${\text{SFG}}_{{\text{ME}}_{\text{HP}}}=\frac{(18.36\bullet {\text{EL}}_{f}^{3} - 7.43{\bullet \text{EL}}_{f}^{2} - 10.43\bullet {\text{EL}}_{f} + 137.32)\bullet 50}{{\text{LHV}}_{\text{LNG}}}$$

where ${\text{SFC}}_{{\text{ME}}_{\text{HP}}}$ is the SFC in diesel mode and ${\text{SFG}}_{{\text{ME}}_{\text{HP}}}$ is the SGC in gas mode for 2MEDHP in g/kWh, $i$ is the fuel type, $\text{EL}$ is the MCR as a decimal, *f* is each voyage phase, ${\text{LHV}}_{\text{actual}}$ is the LHV for the fuel type in use, and ${\text{LHV}}_{\text{LNG}}$ is the LHV of the LNG fuel in MJ/kg both shown in Table 3.

**Table 3:** Lower and higher heating values for RO, DO, and LNG

	RO	DO	LNG
Lower heating value (MJ/kg)	40.2^a^	42.7^a^	50.0^e^
Higher heating value (MJ/kg)	43.0^b^	45.6^c^	53.6^d^

^a^International Maritime Organization (2016b)

^b^SNAME (1961)

^c^Lam (2017)

^d^International Gas Union (2022)

^e^ISO (2016)

The way of calculating the SPFC for 2MEDHP is shown in Eq. 6.

6 $${\text{SPFC}}_{{\text{ME}}_{\text{HP}}}=\frac{\left(-11.99\bullet {\text{EL}}_{f}^{3}+38.30{\bullet \text{EL}}_{f}^{2} - 43.33\bullet {\text{EL}}_{f} + 22.95\right)\bullet 42.7}{{\text{LHV}}_{\text{actual},i}}$$

where ${\text{SPFC}}_{{\text{ME}}_{\text{HP}}}$ is the SPFC for 2MEDHP in gas mode, in g/kWh, $i$ is the fuel type, $\text{EL}$ is the MCR as a decimal, $f$ is each voyage phase, and ${\text{LHV}}_{\text{actual}}$ is the LHV for the fuel type in use in MJ/kg as shown in Table 3.

The SPFC in diesel mode is already considered in Eq. 5.

#### SFC for two-stroke dual engines with gas injection at low pressure

This type of engine operates in diesel and gas mode as well; the way of calculating the SFC in diesel and gas mode is shown in Eqs. 7 and 8, respectively.

7 $$\text{SF}{{\text{C}}_{\text{ME}}}_{\text{LP}}=\frac{\left(75.09\bullet {\text{EL}}_{f}^{3}- 107.06\bullet {\text{EL}}_{f}^{2}+ 28.42\bullet {\text{EL }}_{f}+ 188.98\right)\bullet 42.7}{{\text{LHV}}_{\text{actual},i}}$$

8 $$\text{SG}{{\text{C}}_{\text{ME}}}_{\text{LP}}=\frac{(6.22\bullet {\text{EL}}_{f}^{3}+3.07{\bullet \text{EL}}_{f}^{2} - 32.76\bullet {\text{EL}}_{f} + 168.84)\bullet 50}{{\text{LHV}}_{\text{LNG}}}$$

where ${\text{SFC}}_{{\text{ME}}_{\text{LP}}}$ is the SFC in diesel mode and ${\text{SGG}}_{{\text{ME}}_{\text{LP}}}$ is the SGC in gas mode for 2MEDLP in g/kWh, $i$ is the fuel type, $\text{EL}$ is the MCR as a decimal, $f$ is each voyage phase, ${\text{LHV}}_{\text{actual}}$ is the LHV for the fuel type in use in MJ/kg, and ${\text{LHV}}_{\text{LNG}}$ is the LHV of the LNG fuel in MJ/kg as shown in Table 3.

This type also uses pilot fuel, and Eq. 9 shows how it is calculated.

9 $$\text{SPF}{{\text{C}}_{\text{ME}}}_{\text{LP}}=\frac{\left(3.47{\bullet \text{EL}}_{f}^{2} - 6.55\bullet {\text{EL}}_{f} + 3.64\right)\bullet 42.7}{{\text{LHV}}_{\text{actual},i}}$$

where ${\text{SPFC}}_{{\text{ME}}_{\text{LP}}}$ is the SPFC for the 2MEDHP in gas mode, in g/kWh, $i$ is the fuel type, $\text{EL}$ is the MCR as a decimal, $f$ is each voyage phase, and ${\text{LHV}}_{\text{actual}}$ is the LHV for the fuel type in use in MJ/kg as shown in Table 3.

The SPFC in diesel mode is already considered in Eq. 8.

#### SFC for four-stroke dual engines with gas injection

These types of engines are normally part of the DFDE propulsion systems. On that arrangement, the four-stroke engines with gas injection are coupled to the electrical grid onboard to supply propulsion and the auxiliary energy demanded onboard. The SFC formula for diesel and gas mode is shown in Eqs. 10 and 11, respectively.

10 $${\text{SFC}}_{\text{DDG}}=\frac{\left(-83.93\bullet {\text{EL}}_{f}^{3} + 263.02\bullet {\text{EL}}_{f}^{2} - 261.84\bullet {\text{EL }}_{f}+ 274.6\right)\bullet 42.7}{{\text{LHV}}_{\text{actual},i}}$$

11 $${\text{SGC}}_{\text{DDG}}=\frac{\left(152.18\bullet {\text{EL}}_{f}^{2}-284.77\bullet {\text{EL}}_{f}+285.87\right)\bullet 50}{{\text{LHV}}_{\text{LNG}}}$$

where ${\text{SFC}}_{\text{DDG}}$ is the SFC in diesel mode and ${\text{SGC}}_{\text{DDG}}$ is the SGC in gas mode for 4-stroke engines with gas injection in g/kWh, $i$ is the fuel type, $\text{EL}$ is the MCR as a decimal, $f$ is each voyage phase,${\text{LHV}}_{\text{actual}}$ is the LHV for the fuel type in use in MJ/kg, and ${\text{LHV}}_{\text{LNG}}$ is the LHV of the LNG fuel in use in MJ/kg as shown in Table 3.

This engine type under gas mode uses pilot fuel, and it is calculated as shown in Eq. 12.

12 $${\text{SPFC}}_{\text{DDG}}=\left(-6.06\bullet {\text{EL}}_{f}^{3}+25.93{\bullet \text{EL}}_{f}^{2} - 33.29\bullet {\text{EL}}_{f} + 14.56\right)$$

where ${\text{SPFC}}_{\text{DDG}}$ is the SPFC for the 4-stroke dual engines with gas injection in gas mode, in g/kWh, $i$ is the fuel type, $\text{EL}$ is the MCR as a decimal, $f$ is each voyage phase, and ${\text{LHV}}_{\text{actual}}$ is the LHV for the fuel type in use in MJ/kg as shown in Table 3.

The SPFC in diesel mode is already considered in Eq. 10.

Often, the information in the databases shows the total energy installed onboard. The electrical propulsion motors in the DFDE arrangement have different maximum power than the total energy available from the dual engines. It is essential for the model to differentiate the electrical energy available for propulsion and for auxiliary systems. In this work, the relation between the percentage of the installed energy used for propulsion and the ship’s deadweight is found in Eq. 13.

13 $${\%}P=112839\bullet\text{DWT}^{0.6465}$$

where $\text{\%}P$ is the percentage of the total installed power used for propulsion and DWT is the deadweight of the ship in tons.

#### SFC for steam plants

The GHG study from IMO used a constant SFC for steam plants, independently from the operational load. As occurs with diesel engines, the SFC of steam plants also has a polynomial fit depending on the MCR; this is observed by analyzing the energy balances.

The fuel and gas consumption shown in the energy balances is referenced to the fuel higher heating value (HHV) of 43.032 MJ/kg (SNAME 1961). In this model, it has been separated the amount of fuel required for propulsion and the amount of fuel for auxiliary systems (see the “SFC for turbogenerators” section).

The SFC for propulsion under diesel and gas mode is shown in Eqs. 14 and 15, respectively.

14 $${\text{SFC}}_{T}=\frac{\left(234.13\bullet {\text{EL}}_{f}^{2}-468.56\bullet {\text{EL}}_{f}+523.78\right)\bullet 43.03}{{\text{HHV}}_{\text{actual},i}}$$

15 $${\text{SGC}}_{T}=\frac{\left(202.18\bullet {\text{EL}}_{f}^{2}-432.21\bullet {\text{EL}}_{f}+537.53\right)\bullet 43.03}{{\text{HHV}}_{\text{LNG}}}$$

where ${\text{SFC}}_{T}$ is the SFC in diesel mode and the ${\text{SGC}}_{T}$ is the SGC in gas mode for the conventional steam plants in g/kWh, $i$ is the fuel type, $\text{EL}$ is the MCR as a decimal, $f$ is each voyage phase, ${\text{HHV}}_{\text{actual}}$ is the HHV for the fuel type in use, and ${\text{HHV}}_{\text{LNG}}$ is the HHV for the LNG in use in MJ/kg as shown in Table 3.

For the most modern designs of steam plants on LNGC, the main boilers produce steam at higher pressures and temperatures and include other modifications in the steam plants, improving the efficiency of the steam plant. This type of steam plant is known as ultra-steam plant (MHI 2007).

The specific consumption of the ultra-steam plants under diesel and gas mode is shown in Eqs. 16 and 17, respectively.

16 $${\text{SFC}}_{\text{TU}}=\frac{\left(313.31\bullet {\text{EL}}_{f}^{2}-533.44\bullet {\text{EL}}_{f}+479.83\right)\bullet 43.03}{{\text{HHV}}_{\text{actual},i}}$$

17 $${\text{SGC}}_{\text{TU}}=\frac{\left(352.76\bullet {\text{EL}}_{f}^{2}-593.98\bullet {\text{EL}}_{f}+520.97\right)\bullet 43.03}{{\text{HHV}}_{\text{LNG}}}$$

where ${\text{SFC}}_{\text{TU}}$ is the SFC in diesel mode and ${\text{SGC}}_{\text{T}}$ is the SGC in gas mode for the ultra-steam plants in g/kWh, $i$ is the fuel type, $\text{EL}$ is the MCR as a decimal, $f$ is each voyage phase, ${\text{HHV}}_{\text{actual}}$ is the HHV for the fuel type in use in MJ/kg, and ${\text{HHV}}_{\text{LNG}}$ is the HHV for the LNG in use in MJ/kg both shown in Table 3.

### Fuel consumption for auxiliary engines

The three main auxiliary engine types on LNGC are 4-stroke diesel engines, turbogenerators on LNGC with steam turbines (Mrzljak et al. 2017), and 4-stroke dual engines with gas injection (Zoolfakar et al. 2014; Ekanem and Bucknall 2015; Stoumpos et al. 2018), in addition to the shaft generators when installed.

The fuel consumption of the auxiliary engines is calculated as shown in Eq. 18.

18 $${\text{FC}}_{\text{DG},i}=\sum_{f=1}^{f}\frac{({\text{SFC}}_{\text{DG},f,i,m}+{\text{SPFC}}_{\text{DG},f,i,m})\bullet {P}_{f}\bullet E{L}_{f}\bullet \frac{{N}_{o,f}}{{N}_{a}}{\bullet h}_{f}}{{10}^{6}}$$

where ${\text{FC}}_{\text{DG},i}$ is the annual fuel consumption for auxiliary engines in tons, *i* is the fuel type, $f$ are the voyage phases, $\text{SFC}$ and SGC are in g/kWh, $\text{SPFC}$ is the gas mode in g/kWh, *m* is the engine type, $P$ is the engine power for each phase as kW, EL is the MCR as a decimal, ${N}_{o}$ are the number of engines in operation (Table 1), ${N}_{a}$ is the number of engines installed on board, and $h$ is the hours in operation.

#### SFC for 4-stroke diesel engines

For the 4-stroke diesel engines, the SFC base has been used in the 3rd GHG IMO study for 225 g/kWh when RO fuels are in use and 215 g/kWh when DO fuels are in use (Smith et al. 2015). Then, Eq. 3 is applicable for this type of auxiliary engine.

#### SFC for 4-stroke dual engines with gas injection

This type of engine is the technology selected to supply auxiliary power to the LNGC where the propulsion system is a two-stroke dual engine or DFDE.

The average propulsion power for conventional LNGC (excluding LNG bunker ships) ranges from 23 to 26 MW, and the total installed power ranges from 36 to 40 MW (IHS Markit 2022).

The distribution of the electrical power distribution is shown in Table 4.

**Table 4:** Percentage of energy distributed for auxiliary load and propulsion on DFDE LNGC. Author’s data

Phase	Auxiliary load	Propulsion energy
Normal sailing	12.4%	87.6%
Slow steaming	19.7%	80.3%
Maneuvring	49.1%	50.9%
At anchorage	100%	0%
At port	100%	0%

To calculate the SFC, Eqs. 10–12 are applicable.

#### SFC for turbogenerators

For electrical supply on LNGC with steam plants, there are 2 or 3 turbogenerators (TGs) installed onboard, driven by the steam produced in the main boilers. In addition to these TGs, LNGCs with steam plants have an emergency diesel engine for emergency situations (Fernández et al. 2017). The TGs need a longer time to start than the diesel engines; normally, two TGs in parallel are in operation during maneuvres, in port, and even in normal navigation (Adamkiewicza and Drzewienieckij 2013; Banaszkiewicz 2014; Mrzljak et al. 2018).

The TGs are using the high-pressure and temperature steam produced by the boilers.

Based on the available technical performance sheets, the main boilers use an average of 0.072 and 0.06 kg of fuel to generate 1 kg of steam in diesel mode and gas mode, respectively. The steam consumption in the TGs depends on the load of the steam plant.

The SFC of the TGs for diesel and gas modes is shown in Eqs. 19 and 20, respectively.

19 $${\text{SFC}}_{\text{TG}}=\frac{\left(212.95\bullet {\text{EL}}_{f}^{2}-317.39\bullet {\text{EL}}_{f}+408.15\right)\bullet 43.03}{{\text{HHV}}_{\text{actual},i}}$$

20 $${\text{SGC}}_{\text{TG}}=\frac{(177.46\bullet {\text{EL}}_{f}^{2}-264.49\bullet {\text{EL}}_{f}+340.13)\bullet \text{43,03}}{{\text{HHV}}_{\text{LNG}}}$$

where ${\text{SFC}}_{\text{TG}}$ is the SFC in diesel mode and ${\text{SGC}}_{\text{TU}}$ is the SGC in gas mode for the TGs in g/kWh, $i$ is the fuel type, $\text{EL}$ is the MCR as a decimal, *f* is each voyage phase, ${\text{HHV}}_{\text{actual}}$ is the HHV for the fuel type, and ${\text{HHV}}_{\text{LNG}}$ is the HHV for the LNG in use in MJ/kg, both shown in Table 3.

The LNGC with ultra-steam plants will be also used in Eqs. 19 and 20.

### Fuel consumption for auxiliary boilers

The operational profile of auxiliary boilers is one of the most difficult emission sources to get accurate information (California Air Resources Board 2011; Peeters 2018) and often is left out of many emission inventories (Moreno-Gutiérrez et al. 2012); in the same way, it is very challenging to know the real power output from the boilers (Li 2017).

Fuel consumption from 18 LNGCs has been analyzed to estimate the power output from the auxiliary boilers. Only fuel type and consumption were available. Using a simplistic approach, the power output from the auxiliary boiler for LNGC is calculated by applying Eq. 21.

21 $${P}_{B}=\frac{{\text{FC}}_{{B}_{i}}\bullet {\text{LHV}}_{i}\bullet \frac{{\varepsilon }_{B}}{100}}{3600}$$

where ${P}_{B}$ is the power output from the auxiliary boiler in kW, ${\text{FC}}_{B}$ is the fuel consumption of the auxiliary boiler in kg/h, ${\varepsilon }_{B}$ is the boiler efficiency assumed constant (84%) (Karthikeyan 2014), $i$ is the fuel type, and $\text{LHV}$ is the LHV for the fuel type in use in MJ/kg as shown in Table 3. It is assumed that the auxiliary boilers only use RO or DO fuels. Table 5 shows the power output and time in operation used in this model.

**Table 5:** Operational time and output power from auxiliary boilers. Author’s data

Voyage phase	%Time in operation	Power output (kW)
Normal navigation	0	0
Slow steaming	0	0
Maneuvring	75.6	623
At anchorage	91.4	1,057
At berth	79.0	980

Data shows that during normal navigation and slow steaming, the auxiliary boilers were in operation 42% of the time. However, it uses the same assumption as IMO does in regards to the efficient operation onboard, so as a general rule, the auxiliary boiler will remain off during navigation (Myśków and Borkowski 2012; Smith et al. 2015).

From Eq. 21, the SFC might be obtained by reverse engineering from the power output calculated, getting 100 g/kWh as the average SFC. This is not very accurate because it assumes constant fuel LHV and boiler efficiency, due to this, so it has been decided to use constant SFC 305 g/kWh for RO and 295 g/kWh for DO (Smith et al. 2015).

The fuel consumption for the auxiliary boilers can be calculated as shown in Eq. 22.

22 $${\text{FC}}_{\text{AB},i}=\sum_{f=1}^{f}\frac{{\text{SFC}}_{i,f}\bullet {P}_{f}\bullet {h}_{f}}{{10}^{6}}$$

where ${\text{FC}}_{\text{AB},i}$ is the annual fuel consumption in tons, $i$ is the fuel type, $f$ are the voyage phases, $\text{SFC}$ is the SFC in g/kWh, $P$ is the engine power for each phase as kW, and $h$ is the number of hours in operation.

### LNG burnt in the gas combustion unit

Natural gas undergoes a transformation into a liquid state at cryogenic temperatures, approximately − 163 °C, facilitating its transportation via LNG carriers (LNGCs) (Kurle et al. 2015; Migliore et al. 2015). However, these cargo tanks are not perfectly insulated, allowing heat to seep inside and raise the internal temperature. This process leads to the generation of boil-off gas (BOG), with the extent of BOG generation strongly influenced by the design of the cargo tanks (Chang et al. 2008). BOG can serve various purposes, including use as fuel, reliquefication and return to the cargo tanks, or incineration in a gas combustion unit (GCU) (Dimopoulos et al. 2016).

Given that BOG generation is inevitable, it must be effectively managed, prioritizing safety and efficiency (Chang et al. 2008; Dimopoulos and Frangopoulos 2008; Zanne and Grčić, 2009; Kim et al. 2019). LNGCs equipped with steam plants channel BOG to the main boilers for combustion, subsequently producing excess steam that is condensed in the main condenser. Conversely, LNGCs featuring 2- or 4-stroke conventional diesel engines cannot utilize BOG as fuel. Therefore, these vessels are equipped with reliquefication plants and/or GCUs (Sinha et al. 2012). It is worth noting that reliquefication plants can also be found on LNGCs employing other propulsion systems, although this is less common (Kurle et al. 2015; Sinha et al. 2012; Tu et al. 2019).

The GCU is always found on board except on LNGC with steam plants.

In this proposed method, it is assumed that the GCU is installed on LNGC with dual fuel engines (2 and 4 strokes) (Fernández et al*.* 2017), while on other LNGCs, the BOG is guessed to be managed by the auxiliary boilers and/or reliquification plants.

To estimate the consumption of BOG in the GCU (Table 6), it has been analyzed the operational data of 24 LNGCs with GCU on board.

**Table 6:** GCU consumption and operational time for each voyage phase. Author’s data

Propulsion type	Voyage phase	%Time GCU is ON	Average BOG burnt in GCU (ton/h)
4-stroke dual engines	Normal navigation	12.5%	0.222
	Slow steaming	32.0%	0.662
	Average navigation	23.5%	0.483
	Maneuvring	29.7%	1.090
	At anchorage	53.6%	1.261
	At berth	80.8%	1.001
2-stroke dual engines	Normal navigation	13.4%	0.323
	Slow steaming	14.5%	0.324
	Average navigation	14.1%	0.339
	Maneuvring	40.7%	0.731
	At anchorage	8.4%	0.305
	At berth	83.4%	0.787

The normal practice of LNGC is to minimize the waste of BOG to avoid misspend of energy. Usually, during navigation, the optimal and what ship operators are looking for is to evade running the GCU; however, the data shows that GCU is in operation for a considerable time where the ships are sailing. This can be explained because sometimes the LNG is loaded on board ships at high pressures and temperatures, being necessary to burn excess gas in the GCU during navigation to maintain pressure in cargo tanks.

In this model, it is assumed that only LNG vessels with DFDE and with dual two-stroke engines use the GCU. To calculate the BOG burnt in the GCU, Eq. 23 is used.

23 $${\text{FC}}_{\text{GCU}}=\sum_{f=1}^{f}\frac{{\text{BOGH}}_{f}\bullet {\text{Op}}_{f}\bullet {h}_{f}}{{10}^{6}}$$

where ${\text{FC}}_{\text{GCU}}$ is the BOG consumed per year in tons, $\text{BOGH}$ is the BOG consumption per hour in ton/h, $\text{Op}$ is the percentage of time that the GCU is in operation as shown in Table 6, $f$ are the voyage phases, and $h$ is the hours in operation.

### Operational profile

It is assumed that at anchorage or at port, the main engine is off, in line with Olmer et al. (2017). The main engine load profile for this model is shown in Table 7, while the auxiliary engine load and number of engines in operation are shown in Table 1. Besides, it has been extracted the energy demand for each phase as shown in Table 8.

**Table 7:** Main engine load for each voyage phase. Author’s data

Voyage phase	Turbine	DFDE*	2-stroke diesel	2-stroke dual
Normal navigation	79.2%	70.7%	73.3%	78.0%
Slow steaming	43.5%	60.0%	45.3%	40.5%
Average navigation	65.4%	64.6%	51.6%	51.1%
Maneuvring	11.5%	40.4%	10.2%	14.6%
At anchorage	0%	0%	0%	0%
At berth	0%	0%	0%	0%

^*^On ships with DFDE propulsion, dual four-stroke engines have connected alternators that produce electrical power. This electrical energy is used to supply the energy required for propulsion, and for auxiliary energy, for this reason, the MCR is higher in maneuvring, at anchorage, and at port than the other propulsion systems. However, it was observed that in LNG ships with this propulsion system, if we observe the MCR of the propulsion system in isolation (power delivered to the propeller), the MCR values were 80.5% for “Normal Navigation,” 41.4% for “Slow Steaming,” 10.2% for “Maneuvring”, 3.2% for “Anchored,” and 1.2% for “Port”

**Table 8:** Demanded auxiliary load for each voyage phase and propulsion system. Author’s data

Voyage phase	Turbine (kW)	DFDE* (kW)	2-stroke diesel (kW)	2-stroke dual (kW)
Normal navigation	1,620	2,793	6,046	3,303
Slow steaming	1,540	2,624	6,092	3,357
Average navigation	1,571	2,988	6,072	3,345
Maneuvring	1,500	2,304	6,059	3,321
At anchorage	1,820	2,481	6,391	3,562
At berth	1,960	2,599	4,962	3,783

^*^On ships with DFDE propulsion, dual four-stroke engines have connected alternators that produce electrical power. This electrical energy is used to supply the energy required for propulsion, and for auxiliary energy, for this reason, the MCR is higher in maneuvring, at anchorage, and at port than the other propulsion systems. However, it was observed that in LNG ships with this propulsion system, if we observe the MCR of the propulsion system in isolation (power delivered to the propeller), the MCR values were 80.5% for “Normal Navigation,” 41.4% for “Slow Steaming,” 10.2% for “Maneuvring”, 3.2% for “Anchored,” and 1.2% for “Port”

#### Fuel mix

The fuel type in use on board, the LNGC is influenced by the fuel prices, LNG prices, and trading area. Tables 9 and 10 show the fuel mix for LNGC. For this model, it has been assumed that the auxiliary boilers only use RO and DO fuels. This assumption has been made despite the authors’ awareness that LNG can be used by the auxiliary boilers; but since this alternative has not been seen in the HFD dataset analyzed, then it has been decided to omit LNG as a fuel alternative for auxiliary boilers.

**Table 9:** Fuel mix (%) on LNGC (excl. aux boiler) for different voyage phases. Author’s data

Voyage phase	Turbine			DFDE			2-stroke diesel			2-stroke dual		
	RO	DO	Gas	RO	DO	Gas	RO	DO	Gas	RO	DO	Gas
Normal navigation	22.9	0.5	76.6	14.3	1.5	84.2	99.4	0.6	0	12.9	2.8	84.5
Slow steaming	17.7	0.5	81.8	15.3	2.0	82.7	98.6	1.4	0	13.1	3.5	83.6
Average navigation	19.7	0.5	79.8	14.1	1.8	84.2	98.8	1.2	0	12.9	3.3	84.0
Maneuvring	26.7	1.2	72.2	36.9	4.9	58.2	84.4	15.6	0	12.2	10.3	78.0
At anchorage	35.4	3.1	61.5	37.3	16.5	46.2	64.6	35.4	0	12.5	28.3	59.8
At berth	35.4	3.8	60.8	35.5	21.2	43.3	56.3	43.7	0	12.4	21.6	66.7
**Total**	**23**	**1**	**76**	**23**	**6**	**71**	**85**	**15**	**0**	**13**	**12**	**76**

**Table 10:** Fuel mix (%) on LNGC for aux boilers for different voyage phases. Author’s data

Voyage phase	Turbine		DFDE		2-stroke diesel		2-stroke dual	
	RO	DO	RO	DO	RO	DO	RO	DO
Normal navigation	0	0	97.9	2.1	99.4	0.6	82.2	17.8
Slow steaming	0	0	97.3	2.7	98.6	1.4	78.9	21.1
Average navigation	0	0	97.5	2.5	98.8	1.2	79.6	20.4
Maneuvring	0	0	95.7	4.3	80.5	19.5	54.2	45.8
At anchorage	0	0	91.9	8.1	70.5	29.5	30.6	69.4
At Berth	0	0	90.3	9.7	56.3	43.7	36.5	63.5
**Total**	**0**	**0**	**95.8**	**4.2**	**85**	**15**	**60**	**40**

### Atmospheric emissions

In this model, the emissions are calculated using a fuel-based approach. This is expressed in Eq. 24.

24 $${E}_{j}=\sum_{f=1}^{f}{\text{FC}}_{i,ES,f}\bullet {\text{EF}}_{\text{j}}$$

where $\text{E}$ is the annual emissions for each ship in tons, $j$ is the atmospheric emission (CO_2_, SO_x_, NO_x_, etc.), $\text{FC}$ is the annual fuel consumption for each ship in tons, $i$ is the fuel type, $f$ are the voyage phases, $\text{ES}$ are the emission sources on board, and the $\text{EF}$ are the emission factor.

The emission factors for some pollutants are applied straightforwardly, with one emission factor (a ton of pollutant per ton of fuel) for each fuel type to obtain the tones of the pollutant (Table 11).

**Table 11:** Emission factors (fuel-based) for CO_2_, CO, CH_4_, N_2_O, NMVOC, and PM. (Smith et al. 2015)

Fuel type	CO_2_	CO	CH_4_	N_2_O	NMVOC	PM
RO	3.114	0.00277	0.00006	0.00016	0.00308	0.00728
DO	3.206	0.00277	0.00006	0.00015	0.00308	0.00097
LNG	2.750	0.00783	0.05120	0.00011	0.00301	0.00018

There are three more pollutants that are considered in this work: NO_x_, SO_2_, SO_4_, and black carbon, and the methods used to calculate them are explained in the following subsections.

#### NO_x_ emissions

The 3rd IMO GHG Study had a high uncertainty level in regards to NO_x_ emissions based on fuel, especially when LNG is used as fuel. The 4th IMO GHG Study proposed to use an energy-based method to calculate the NO_x_ emissions.

In this model, to be standardized with the other pollutants, the fuel-based approach is used knowing the uncertainties associated with this approach.

The emission factor for each fuel type and engine type is shown in Table 12.

**Table 12:** NO_x_ emission factors. (Smith et al. 2015)

Engine type (tier)	Building year	Emission factor (ton NOx/ton fuel)		
		RO	DO	LNG
SSD (Tier 1)	Built in or before 2000	0.09282	0.08725	0.00783
SSD (Tier 2)	Built in or after 2011	0.08718	0.08195	0.00783
SSD (Tier 3)	Built in or after 2016	0.07846	0.07375	0.00783
MSD (Tier 1)	Built in or before 2000	0.06512	0.06121	0.00783
MSD (Tier 2)	Built in or after 2011	0.06047	0.05684	0.00783
MSD (Tier 3)	Built in or after 2016	0.05209	0.04896	0.00783

For the auxiliary boilers, the NOx factor is assumed 0.02 t NOx/t fuel (Smith et al. 2015).

#### SO_x_ emissions

The SOx emissions are directly influenced by the fuel quality. The SOx emissions are calculated as per Eq. 25.

25 $${E}_{{\text{SO}}_{\text{x}}}={\sum }_{ES}{\text{FCA}}_{i,ES}\bullet {S}_{i}$$

where ${E}_{{\text{SO}}_{\text{x}}}$ is the SO_x_ emissions in tons, $\text{FCA}$ is the annual fuel consumption of all emission sources in tons, ES are the emission sources on board, $i$ is the fuel type, and $S$ is the sulfur content on each fuel type (see Table 13).

**Table 13:** Assumed sulfur content on different fuel types. (Smith et al. 2015)

Type of fuel	Sulfur content (%)
RO	0.5^a^
DO	0.1
LNG	0

^a^Based on the maximum sulfur content allowed on residual oil without any exhaust gases cleaning system on board

Approximately 98% of the sulfur is transformed into gaseous SO_2_, and 2% is converted into particle emissions, SO_4_ (Cooper 2002; Smith et al. 2015).

#### Black carbon emissions

Black carbon (BC) emissions are of significant concern because they consist of aerosols that absorb solar radiation and convert it into heat, as noted by Messner (2020). It is important to recognize that reducing fuel sulfur content may inadvertently amplify the global warming effect of BC and increase human exposure to elemental carbon particles due to their extended atmospheric lifetime, as discussed by Lack and Corbett (2012). Therefore, the evaluation and control of BC emissions hold substantial importance, given their impact on both climate change and human health, as highlighted by Brewer (2019). Notably, BC emissions pose particularly severe consequences in Arctic regions, as emphasized by Zhang et al. (2019).

It is worth noting that BC emissions have not received extensive attention within the maritime industry, making it challenging to establish a consensus regarding their estimation based on the type of fuel used. The prevailing BC emission factor associated with fuel is often cited as 0.35 g BC/kg of fuel, and this value tends to remain constant regardless of the specific fuel utilized, as observed in studies conducted by Corbett et al. (2010), Mjelde et al. (2014), and Winther et al. (2014).

In this model, the BC emission factors are shown in Table 14, proposed by the International Council of Clean Transport (ICCT).

**Table 14:** BC emission factors. ICCT, (2017)

Engine type	Fuel type	BC emissions (g BC/kg fuel)
2-stroke diesel engine	RO	$0.0382\bullet {\text{EF}}_{e}^{-0.392}$
	DO	$0.0072\bullet {\text{EF}}_{e}^{-0.557}$
	LNG (90% reduction from RO)	($0.0382\bullet {\text{EF}}_{e}^{-0.392}$)$\bullet 0.1$
4-stroke diesel engine	RO	$0.0509\bullet {\text{EF}}_{e}^{-0.978}$
	DO	$0.0243\bullet {\text{EF}}_{e}^{-1.167}$
	LNG (90% reduction from RO)	$(0.0509\bullet {\text{EF}}_{e}^{-0.978}$)$\bullet 0.1$
2-stroke dual engine	RO	8.4% of PM emissions
	DO	8.4% of PM emissions
	LNG (90% reduction from RO)	8.4% of PM emissions
4-stroke dual engine	RO	8.4% of PM emissions
	DO	8.4% of PM emissions
	LNG (90% reduction from RO)	8.4% of PM emissions

The boilers were not included in the ICCT report, so for RO and DO, the emission factors used are the ones explained in Corbett et al.’s (2010), Mjelde et al.’s (2014), and Winther et al.’s (2014) study; a full overview is compiled in Table 15.

**Table 15:** BC emission factor fuel-based for boilers

Emission source	Fuel type	BC emissions (g BC/kg combustible)
Boilers	RO	0.35
	DO	0.35
	LNG (90% reduction from RO)	0.035

## Model validation

In this research, we validate our methodology, referred to as “LNGCSP,” by applying it to the LNGC fleet and comparing it with the method utilized in the 3rd Study of GHG conducted by IMO. We assess the accuracy of fuel consumption variables obtained through both methods by comparing them with annual consumption data collected from ship performance monitoring systems aboard 73 LNGCs. To evaluate the models, we employ the “*t*-student” statistic, which determines if there is a significant difference between the means of the two samples. In our case, one sample comprises the models obtained using the two methodologies for estimating fuel consumption, while the other consists of the actual data measured aboard the LNGCs. Furthermore, we calculate and analyze the Pearson correlation coefficient (“*p*”) as outlined in Eq. 26 (Peña-Troncoso et al. 2019).

26 $${p}_{X,Y}=\frac{{\sigma }_{XY}}{{\sigma }_{X}\bullet {\sigma }_{Y}}$$

where ${\sigma }_{XY}$ is the covariance of (*X*, *Y*), ${\sigma }_{X}$ is the standard deviation of *X*, and ${\sigma }_{Y}$ is the standard deviation of *Y*.

In the “*t*-student” statistic, the dependent variable is the difference between the means of two samples: one from the fuel consumption data estimated by the two methods (IMO and LNGCSP) and the other from actual measured data. Although fuel consumption data itself is not normally distributed, the differences between means tend to follow a normal distribution, especially with a large sample size of 73 LNGCs, according to the central limit theorem. The two-tailed *t*-test is used because it is robust to moderate deviations from normality and variance assumptions, and it requires normality only for the differences in means. Unlike some variations of this method, a two-sample test does not presuppose a specific direction of results, and in our research paper, we do not assume a priori whether the results will indicate overestimation or underestimation.

In our analysis, we established a significance level of 0.05. The null hypothesis assumes no significant difference between the means of the two samples. For the LNGCSP model, the null hypothesis was not rejected for RO, LNG, and CO_2_ emissions, indicating no significant difference from the actual data. However, the null hypothesis was rejected for DO consumption, where the IMO model showed better performance.

One notable advantage of this test is that it does not rely on expert judgment, and its results are widely accepted within the scientific community (Habibnia et al. 2020). Consequently, the “*t*-student” test is a valid and recognized method for comparing samples in scientific experiments.

### Comparison of methods

The 3rd IMO GHG Study reported a total of 46 Mtons of CO_2_ emissions for gas carriers (LNG and LPG combined). As a starting point, we applied the methodology outlined in the 3rd IMO GHG Study to assess the accuracy of its underlying assumptions. Our verification process included establishing a threshold of + 5% to ensure that the assumptions remained within reasonable levels of accuracy. The result obtained after reproducing the IMO method was 47.5 Mtons of CO_2_, a 3% deviation from the result reported in the study; therefore, it can be assumed that the application of the method from IMO is within an acceptable range of accuracy.

Next, we compared the IMO and LNGCSP models against real data. This comparison aims to show which of the two models reflects the reality of LNGCs more accurately, analyzing which of the two models is closer to the actual data reported by the ships (Van Nieuwkoop et al. 2013). The 73 LNGCs are categorized as shown in Table 16.

**Table 16:** Categorization of the LNGC used in the comparison by propulsion system. Author’s data

Propulsion type	No. ships	Propulsion type	No. ships
2-stroke diesel engine	5	2-stroke dual engine	2
Steam turbine	45	4-stroke dual engine	20

The results obtained (Table 17) are evaluated with 1 degree of freedom by applying the concept of maximum likelihood and hypothesis testing of the *p*-value (Montenegro et al. 2001; Lubiano et al. 2017; Trede 2020). The null hypothesis assumes no significant difference between the means of the two models (IMO and LNGCSP) and the actual data. A lower *p*-value indicates that the results of the evaluated model differ from the real data, while a higher *p*-value indicates that the model is closer to the real data. Therefore, the model with higher *p*-values is the closest to reality and can be assumed to be more accurate.

**Table 17:** Pearson correlation coefficient (p) for the variables compared for each method. Author’s data

Variables	IMO		LNGCSP	
	*p*-value	Statistical	*p*-value	Statistical
Annual RO consumption	0.0013	− 3.2786	0.1856	− 1.3304
Annual DO consumption	0.4786	0.7105	0.0228	2.3022
Annual LNG consumption	0.0051	− 2.8453	0.9209	0.0995
Annual CO_2_ emissions	$5.14 \times {10}^{-6}$	− 4.7453	0.9412	0.0739

**Fig. 3:**
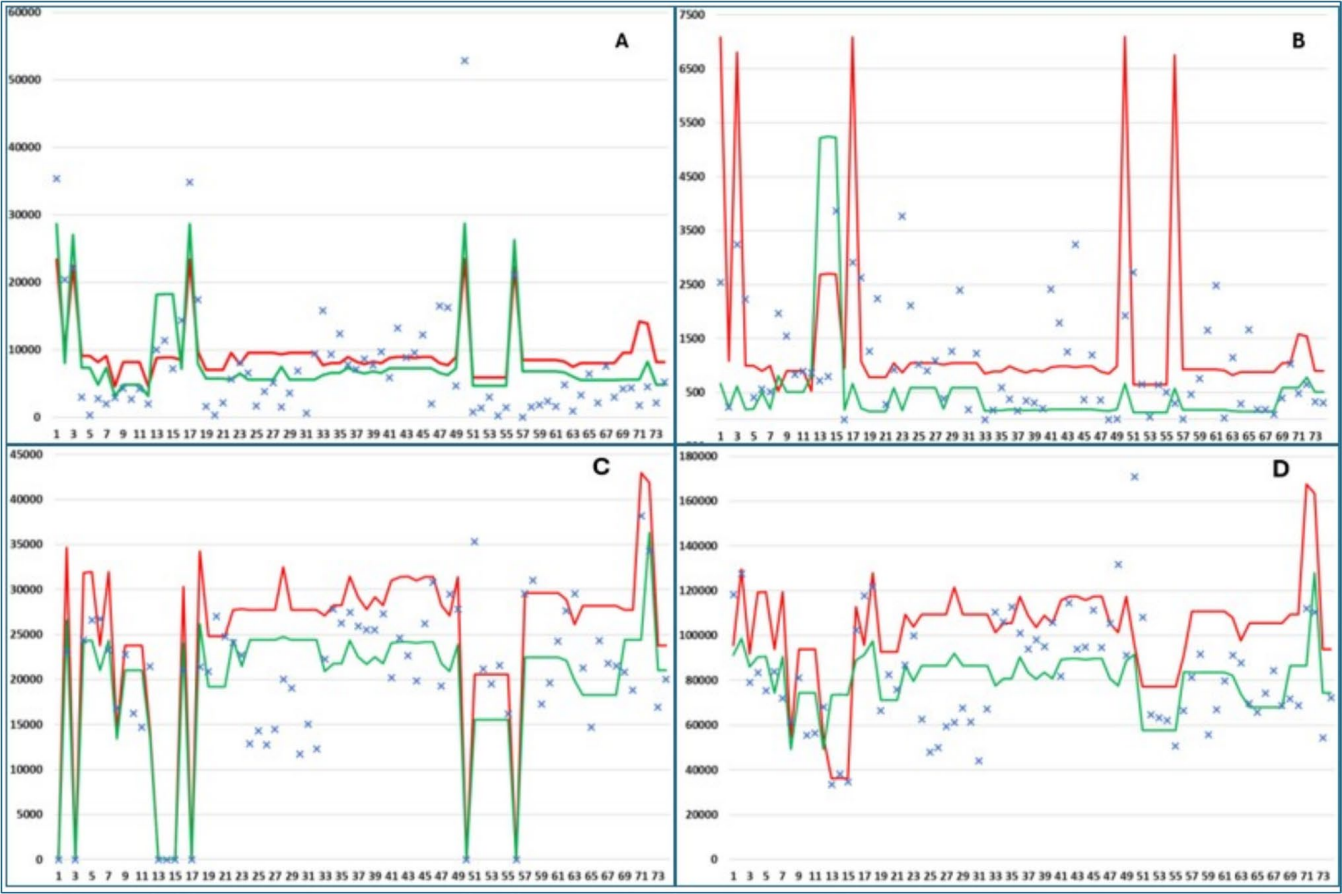
Comparison of models versus annual results of each LNG carrier. Y-axis is in tons. **A** RO consumption, **B** DO consumption, **C** LNG consumption, **D** CO_2_ emissions. Blue cross: ship’s annual result. Green line: LNGCSP model prediction. Red line: IMO model

Although the LNGCSP model performed better (Fig. 3) for most fuel consumption variables (RO, LNG, and CO2), the IMO model showed better performance for DO consumption, where the null hypothesis was rejected.

Table 17 shows that the LNGCSP model is closer to reality in estimating the consumption of RO, LNG, and CO_2_ emissions, showing higher deviation versus real data for DO consumption.

## Future work

There are two principal areas where this model shall be further developed, auxiliary boilers and GCU, to consider the LNG consumed by the auxiliary boilers and the GCU usage on other LNGCs apart from the LNGC with DFDE and 2-stroke dual engines.

## Conclusions

This study has established a comprehensive suite of algorithms to determine fuel consumption across various engine types and consumers onboard LNGCs. The model presented herein forms a robust foundation for future LNGC modeling and simulation efforts, effectively capturing critical complexities related to operational efficiency and emission inventories.

A direct comparison of fuel consumption between the IMO and LNGCSP models, alongside real consumption data from 73 LNGCs, demonstrates that the model developed in this paper outperforms the methodology employed by the IMO in their 3rd GHG Report, except for the DO consumption, where the IMO model shows better performance. Companies operating LNGC can directly apply these insights to more accurately forecast fuel consumption and optimize fuel efficiency across various engine types, leading to both economic and environmental benefits.

The analysis of *p*-values derived from the “*t*-student” test, which evaluates model performance against actual data collected from the 73 LNGCs in service, strongly affirms that the model developed in this research consistently generates values closely aligned with real-world LNGC fuel consumption (including RO, LNG) and CO_2_ emissions. However, the divergence between the IMO method and actual observations is notably reduced only for DO consumption.

Furthermore, the use of real data from the 73 operational LNGCs has resulted in a precise operational profile. This outcome is a direct result of this research and provides valuable insights for addressing future gaps in LNGC-specific modeling.

## Data Availability

This is not applicable to this paper.

## Acronyms

AER: Annual efficiency ratio
BC: Black carbon
BOG: Boil-off gas
DFDE: Dual fuel diesel electric
DO: Distillate fuel oil (MDO, MGO)
GCU: Gas combustion unit
GHG: Greenhouse gases
HFD: High-frequency data
HP: High pressure
HHV: Higher heating value
HSD: High-speed diesel
IMO: International Maritime Organization
LHV: Lower heating value
LNG: Liquified natural gas
LNGC: LNG carrier
LNGCSP: Specific model for LNGC developed in this work
LP: Low pressure
LPG: Liquified petroleum gases
MCR: Maximum continuous rating
MSD: Medium-speed diesel
Mtons: Millions of tons
NMVOC: Non-methane volatile organic compounds
PM: Particulate matter
RO: Residual oil (HFO, LFO)
SFC: Specific fuel consumption
SGC: Specific gas consumption
SNAME: Society of Naval Architects and Marine Engineers
SPFC: Specific pilot fuel consumption
SSD: Slow-speed diesel
